# Additive Manufacturing of Bioresorbable Tracheal Stents from PDO/PCL Blends: Effects of Composition and Processing on Structure and Properties

**DOI:** 10.3390/ma19194117

**Published:** 2026-09-26

**Authors:** Carmen Sánchez González, Mauro Malvè, Cristina Díaz Jiménez

**Affiliations:** 1 Asociación de la Industria Navarra. Carretera Pamplona 1, 31191 Cordovilla, Navarra, Spain; cdiaz@ain.es; 2 Departamento de Ingeniería, Universidad Pública de Navarra (UPNA), Av. Cataluña, s/n, 31006 Pamplona, Navarra, Spain; mauro.malve@unavarra.es; 3 Instituto de Materiales Avanzados y Matemáticas (INAMAT2), Public University of Navarre (UPNA), Edificio Jerónimo de Ayanz, Campus de Arrosadía, 31006 Pamplona, Spain; 4 CIBER-BBN, Research Networking in Bioengineering, Biomaterials & Nanomedicine, Av. Monforte de Lemos, 3-5, Pabellón 11, Planta 0, 28029 Madrid, Spain

**Keywords:** 3D print, FDM, FFF, FGF, stent, PDO, PCL

## Abstract

Fused deposition modeling (FDM) is an additive manufacturing (AM) technology that enables the fabrication of complex and patient-specific devices made of biodegradable polymers. The successful implementation of the process requires exhaustive control of the workflow, focusing on material selection, device design, and manufacturing parameters. The aim of this study is to evaluate and compare the printability, processability, and mechanical performance of a polymer blend for the fabrication of customized airway stents using FDM printing, with a focus on its potential industrial scalability. Two FDM manufacturing approaches were evaluated using standard geometries to manufacture a blend of biodegradable polymers, using either filaments or pellets as feedstock. Moreover, the suitability of a blend made of polycaprolactone (PCL) and polydioxanone (PDO) for manufacturing tracheal stents was studied in comparison to their pure forms. Regarding the manufacturing technology, the feeding method itself was not the primary factor affecting the mechanical properties of the printed components, which was directly related to the geometry of the device and the cooling time polymers required. Furthermore, although the PCL/PDO blend showed a slightly higher mean elastic modulus than pure PCL, the resulting material did not exhibit sufficient radial resistance for airway stent applications. The design and development of 3D-printed medical devices require an evaluation of the entire manufacturing workflow. Device geometry and material selection are strongly influenced by the manufacturing technology selected and have a direct impact on the mechanical performance of the final product.

## 1. Introduction

The ability of additive manufacturing (AM) processes to fabricate complex and customized geometries has driven their adoption in a wide range of biomedical applications. Among these, the production of patient-specific devices from biodegradable polymers has emerged as a promising approach for addressing individual clinical needs [1,2]. However, the successful translation of such devices requires careful control of the complete manufacturing workflow, including material selection, device design, processing conditions, and post-processing operations, prior to clinical use.

In general, AM presents two major limitations: industrial scalability and the availability of suitable materials [2,3]. From a manufacturing perspective, the successful industrial adoption of this technology requires the implementation of robust quality control strategies to guarantee the reliability, biocompatibility, reproducibility, and consistency of the final products. Furthermore, bioabsorbable polymers are particularly susceptible to thermal exposure during processing, as high temperatures may induce unwanted reactions, alter polymer chains, and affect degradation behavior [1]. Material extrusion-based 3D printing, also called fused deposition modeling (FDM), is one of the most widely used AM technologies [3]. Since the FDM process relies on melting and depositing material layer by layer to build the desired geometry, the range of printable materials compatible with the human body may initially seem extensive. Nevertheless, it is primarily used with polymeric materials, which are mainly supplied as filaments, reducing the availability. Moreover, this technology does not necessarily require the use of solvents or additives. In addition, medical-grade materials can maintain their high purity without any modification to their composition that could compromise their medical certification. Furthermore, FDM offers significant potential for industrial translation due to its simplicity, cost-effectiveness, and relatively straightforward scalability. The technology enables a smooth transition from laboratory-scale research to patient-specific manufacturing [4]. In addition, devices can be produced directly on-site, for example, in clinics [5].

Although FDM is the general name for this AM, depending on the type of the extruder and the shape of the raw material, two different methods could be distinguished [6] and are studied in this work. Fused filament fabrication (FFF) is the most widely used method, where the input material is available in spools of filament. This system consists of a series of gears that drive the filament through a heated nozzle, depositing the material layer by layer. Despite its simplicity and the low cost of commercial printers, this method requires high-quality filament to prevent printer clogging. Therefore, the feedstock material must have a consistent diameter and hardness to avoid blockage of the gears [6]. On the other hand, in the second technology, called fused granular fabrication (FGF), the material is fed as granulates. Hence, this method is more versatile and enables the use of a wider variety of materials. Moreover, this technology reduces the production time and cost associated with manufacturing filament, as well as avoids a melting cycle [7], which is a valuable feature when discussing biodegradable polymers. However, achieving homogeneous melting and uniform material deposition may be more challenging.

Furthermore, while theoretically any melt-processable material can be deposited through material extrusion, the development of suitable bioresorbable stents remains a key challenge [8]. A stent is an implantable tubular device intended to maintain the patency of a lumen [9], in particular the airways in the present application. Such materials must combine adequate mechanical performance with biocompatibility and controlled degradation behavior. In addition, their biocompatibility must be maintained throughout their service life [10]. In this respect, a comprehensive analysis of the polymer is required, including its printability, ease of processability, cost, and studying the in-service properties achieved after fabrication [2]. As far as the material is concerned, a device intended to maintain airway patency must meet a set of essential requirements to ensure its effectiveness and safety [11]: it must be mechanically robust to mimic the elasticity and support of the tissue it opens as well as biocompatibility [12]. In the case of a bioresorbable device, that is, one that is biocompatible and degrades into the human body after a predetermined time, it should also remain physiologically stable to resist deformation for as long as its therapeutic function is required. As a result, it is crucial to find the composition of a material that meets the mechanical strength, either because the polymer can meet the exact requirements, or because a compound adapted to these requirements is produced. In this regard, it is equally important to identify the optimal material formulation as well as a manufacturing process that is viable at an industrial application. As a possible solution, multi-material additive manufacturing is positioned as a promising alternative [13], integrating more than one mechanical response into one prototype, often by using material blends with appropriate compositions [6].

Regarding materials, polycaprolactone (PCL) and polydioxanone (PDO) are popular materials in the medical sector, mainly used for scaffold fabrication or sutures [14]. Both materials are synthetic aliphatic polyesters [1]. These two materials have been selected for this investigation and are presented as promising alternatives for manufacturing 3D-printed bioresorbable implantable devices, such as tracheal stents, because of their feasibility in terms of biocompatibility, biodegradability, and suitability with FDM technology [15]. PCL is a flexible material with a slow degradation rate. It presents the highest thermal stability with the lowest melting point and excellent mechanical properties and stability [1,15], so it is commonly used to manufacture scaffolds, as its effectiveness in bone tissue engineering has been demonstrated [11,16]. However, its stiffness can limit the ability to maintain airways or blood vessels open under dynamic physiological stress conditions [12]. In contrast, PDO is a colorless, crystalline, and bioresorbable polymer with a short degradation time of weeks via the hydrolytic process [11,14]. Mostly used for sutures or scaffolds, it presents mechanical properties similar to those of collagen or elastin [16], with a favorable balance of flexibility [15,17]. Nevertheless, this material is not as commercially available as PCL and it is more expensive; therefore, less research has been conducted on this material [4]. In our particular case, we combine the advantages of these two synthetic, biocompatible, and semicrystalline polymers, where PDO contributes to mechanical robustness whereas PCL provides flexibility [17]. For this reason, the present study evaluates each material individually (PDO and PCL), as well as a blend of both polymers. A composition of 75 wt.% PCL and 25 wt.% PDO was selected for the intended application. This formulation is expected to retain the flexibility characteristic of PCL while providing sufficient mechanical strength to prevent rupture. In addition, the incorporation of PDO reduces elongation, minimizing premature deformation. Consequently, the blend represents a promising alternative for achieving a more balanced overall performance [10,11]. As a result, the interest in this compound lies in the fact that it could reduce degradation time without losing mechanical properties, making it suitable for applications that require degradation in a shorter timeframe than other well-known blends, such as PLA/PCL, PLLA/PDO, and PLLA/PCL [14,17].

Although no medical devices for airway management have been found to be manufactured with a blend of PDO/PCL, the combination of these two materials for manufacturing medical devices has been investigated. Some authors have manufactured vascular scaffolds, for aortic replacement in rats and carotid replacement in pigs, combining electrospinning and 3D-FDM printing [10,18,19]. Verreck et al. fabricated a nerve guide made of PCL and PDO (95:5 ratio) via melt compression for nerve regeneration [20]. Fathi et al. designed personalized absorbable gastrointestinal stents for intestinal fistulae and perforations of pigs, using a 25:75 PCL:PDO ratio, which was the combination with a higher modulus of elasticity [11]. Similarly, Park et al. created stents using fibers made of two layers (inner core of PCL and outer of PDO) via electrospinning [12]. In terms of machines, Erben et al. modified a conventional FDM printer to process PDO suture thread as feedstock instead of standard filaments of diameter 1.75 mm to 2.85 mm [21]. Among the literature, most authors process these polymers at a research stage, using chemical methods that include solvents or catalysators [10,11]. Nevertheless, given the cost associated with transferring research knowledge on polymer blends to an industrial scale, composites are not widely considered for medical applications.

Thus, this study evaluates both pure materials and a PDO/PCL blend, with the aim of comparing their printability, processability, and mechanical performance for an application requiring high toughness via FDM using 3D printers to fabricate customized airway stents. Two manufacturing approaches were evaluated using standard geometries, with the objective of applying these findings to stent fabrication. The first approach followed the conventional FFF workflow. In this case, both polymers were weighed, mixed manually, and processed into filament, which was used as feedstock into a conventional filament printer. The second approach consisted of direct pellet extrusion, using a FGF printer. The polymers were weighed, melted and mixed, and shredded to be directly fed into the printer hopper and extruded during the printing process. The comparison will assess the influence of the feedstock preparation route on the printability, processability, and final properties of the manufactured stents, while also evaluating the potential advantages of direct pellet extrusion in terms of process simplification, cost reduction, and industrial scalability.

Given the high cost of processing polymer compounds on an industrial scale, this article aims to analyze and define the extrusion, manufacturing, and post-processing techniques and parameters of the PCL/PDO blend to be used in implantable medical devices with tubular geometries, such as tracheal stents, using 3D printing technology.

## 2. Materials and Methods

Materials required a different processing workflow depending on the printer used, varying between filament or pellets. The processes followed are described in Figure 1.

### 2.1. Selection of Materials

Three different material formulations were evaluated in this study: PDO, PCL, and a PDO/PCL blend containing 25 wt.% PDO and 75 wt.% PCL (hereafter referred to as PDOPCL). PDO and PCL were commercially available materials supplied in filament form, specifically developed for 3D printing and widely used in biomedical applications. In particular, the PDO filament was obtained from Lattice Medical (Lattice Services, Loos, France) and the PCL was supplied by 3D4Makers (3D4Makers B.V, Haarlem, The Netherlands). In contrast, the PDOPCL blend was prepared in-house under controlled laboratory conditions prior to processing.

### 2.2. Thermal Characterization of Raw Materials

Differential scanning calorimetry (DSC) was performed to study the thermal properties of PDO and PCL using the device DSC 25 (TA Instruments, New Castle, DE, USA). The information was used to optimize all processing conditions, from drying to extrusion, as it is essential not to exceed the decomposition temperature of the polymers to prevent thermal degradation. According to the manufacturers’ specifications (Table 1), processing temperatures should remain below 250 °C for PDO and 200 °C for PCL to preserve the material and properties. Therefore, DSC was conducted from −80 °C to 180 °C at a speed of 10 °C/min.

### 2.3. Fused Filament Fabrication

For the first manufacturing approach evaluated in this study (see Figure 1), a simple FFF printer was used, using 1.75 mm filament as feedstock. In the case of both PCL and PDO spools, only a drying step was required prior to printing to remove residual moisture and ensure stable processing conditions. In addition, the PDOPCL was also dried after filament was obtained. The drying process was performed using the conventional filament drier Filament Dry Box 2.0 (Creality 3D Technology Co., Ltd., Shenzhen, China), and drying conditions are included in Table 2.

For the preparation of the PDOPCL blend, materials were weighed according to the desired composition using the precision balance Sartorius Acculab Vicon VIC-123 (Acculab, Göttingen, Germany). Then, materials were shredded and granulated using the GP20 Hybrid machine (3Devo B.V., Utrecht, The Netherlands), yielding particles smaller than 4 mm. The shredded and granulated materials were fed directly into the Filament Maker Composer 450 extrusion system (3Devo B.V., Utrecht, The Netherlands) to produce 1.75 mm-diameter filaments suitable for FFF printing. The extrusion system features a 335 mm screw and four independently controlled heating zones (H1–H4, Table 3), allowing precise adjustment of the thermal profile throughout the filament production process. Both fans were set at 100%, and the screw speed was 2 rpm. Finally, filaments were fed into the single-extruder Voladora V3 printer (Tumaker, Indart 3D, Irún, Spain) for printing the specimens.

### 2.4. Fused Granular Fabrication

For the direct pellet extrusion process (see Figure 1), PDO and PCL were weighed using the same precision balance described previously, Sartorius Acculab Vicon VIC-123 (Acculab, Göttingen, Germany), for the blend. As before, materials were shredded and granulated using a 3Devo GP20 Hybrid (3Devo B.V., Utrecht, The Netherlands), obtaining particles smaller than 4 mm. The granulated materials were then melt-blended at 130 °C for 10 min in the AREC.X Heating Magnetic Stirrer (VELP Scientifica Srl, Usmate, Italy) to promote a homogeneous mix based on the temperatures obtained in the DSC (see Section 2.2). Following blending, the blend was again shredded and granulated, and pellets were fed directly into the printer hopper Mini Modular (Tumaker, Indart 3D, Irún, Spain). This printer was equipped with the screw-based extrusion system, V4 Pellet Extruder (Mahor.XYZ, Andosilla, Spain), which featured two independently controlled heating zones, one located at the material inlet and the other at the nozzle outlet, that was equipped with a 0.8 mm nozzle diameter. In addition, the system incorporated a variable-speed screw with a useful length of 50 mm (total length of 80 mm) and diameter of 8 mm, allowing precise control of the material flow during the printing process. The specimens were fabricated using the processing parameters summarized in Table 4.

In this technology, the most critical parameters are the flow rate, which is the speed at which the screw rotates (Table 4), and the extrusion multiplier, which is the volume of material extruded (Table 5). As the software used (3D Slicer) is primarily designed for filament-based printing, calibration of the extrusion flow is not a trivial task and requires adjustment. To this end, and following the manufacturer’s recommendations, a series of flow rate calibration tests were conducted to obtain these parameters experimentally. A calibration coin (10 mm diameter) was used. Both the material flow rate and the extrusion multiplier were systematically adjusted until an optimal deposition condition was achieved. The selected parameters ensured adequate dimensional accuracy and surface quality while avoiding both over-extrusion and under-extrusion defects, as shown in Figure 2.

### 2.5. Specimen Printing

The two FDM printers used to process the materials described in this study were manufactured by the same company and configured with interchangeable printheads. Specifically, the pellet extruder installed on the Mini Modular printer can be exchanged with the filament extruder of the Voladora V3 printer. This configuration enables a direct comparison of the performance of the two extrusion approaches (FFF and FGF) while maintaining the same printer platform and geometry-slicing software. Therefore, in both cases the printing process was prepared using the software Simplify3D^®^, version 5.1.2 (Simplify3D LLC, Cincinnati, OH, USA). For each material, parameters were set individually following the manufacturer’s instructions in the case of the commercial filament and performing different tests until the optimal printing quality was acquired in the case of PDOPCL.

First, to compare both manufacturing techniques, five rectangular and five tubular specimens of PDOPCL were manufactured using the FFF and FGF printing processes. Five rectangular specimens of 40 mm (length) × 5 mm (width) × 2 mm (thickness) and five tubular specimens of diameter 10 mm and height 5 mm were produced for each group. Tensile specimens were not designed in accordance with ISO standards because, as previously demonstrated, the change of section of FDM specimens have stress concentration regions and weak points that directly influence on mechanical properties [22]. Therefore, the specimen length was set to 40 mm, corresponding to the length of the stents used in this study. Moreover, tubular specimens were printed using spiral mode to ensure the most constant material flow possible during extrusion and to avoid retractions. Then, a comparison of the three materials using the same dimensions as the previous test and the FFF printer was performed (Table 6), manufacturing the two different types of samples. This test was performed to determine the baseline characteristics of each material, as well as the performance of the blend. Finally, three tracheal stents of each material composition (PDO, PCL, and PDOPCL) were manufactured using a previously validated stent design with a geometry in X [23]. A length of 40 mm and a diameter of 4.5 mm were selected to fit the rabbit trachea in future in vivo studies carried out by this research group. This final test compared and validated the materials’ performance, as well as the printing process of the designed medical device to keep the airways open.

### 2.6. Mechanical Characterization

Axial tensile and radial compression strength tests were conducted using a Zwick Roell universal testing machine (Zwick GmbH & Co. KG, Ulm, Germany; Figure 3), using a load cell of 10 kN. As described in other studies [22], tensile testing conditions are based on ISO 527-1:2019 Plastic—Determination of tensile properties [24].The specimens had a total length of 40 mm. Five millimeters of each end were clamped in the grips, resulting in an initial gauge length of 30 mm. The specimens were then stretched along their longitudinal axis until failure. Engineering strain was calculated from the crosshead displacement and the initial gauge length. Initially, a 1 MPa preload is applied. The test speed is 50 mm/min and the speed for calculating the tensile modules is 1 mm/min. On the other hand, the radial testing conditions are completed under a cyclic testing program of Zwick Roell based on the ASTM F3067 Standard Guide for Radial Loading of Balloon-Expandable and Self-Expanding Vascular Stents [25], to measure radial compression force on stents and tubular specimens. It consists of squeezing the specimen radially, simulating the mechanical loads acting on the device, while continuously recording force and displacement. In the case of the stent, this method is used to evaluate the compressive strength and radial support provided, which are key factors in maintaining airway patency. Furthermore, the test allows the investigation of structural collapse and permanent deformation resulting from compressive loading. In this work, external diameter is reduced from 10 mm to 7 mm in the case of the tubular specimen and from 4.5 mm to 2.5 mm for stents, at a test speed of 10 mm/min for both tests. Results are reported as mean ± standard deviation (SD), calculated from the experimental replicates described in Section 2.5.

## 3. Results

Before the manipulation of polymers, DSC was performed to study the thermal properties of PDO and PCL. The information was used to optimize all processing conditions, from drying to extrusion. Figure 4 shows the results of the characterization of one of the samples of each material, in which melting temperatures (Tm) are observed as endothermic peaks, corresponding to polymer melting, at around 60 °C for PCL and 105 °C for PDO. Furthermore, it is essential not to exceed the decomposition temperature of the polymers to prevent thermal degradation (see Table 1). According to the manufacturers’ specifications, processing temperatures should remain below 250 °C for PDO and 200 °C for PCL to preserve the material and properties.

In addition, DSC analyses were performed on the blends processed using the different manufacturing technologies. They revealed the influence of blend composition on the area under the melting peaks, with a first peak observed at approximately 60 °C attributed to the melting of the PCL and a second peak with a smaller area due to the lower content of PDO around 105 °C (Figure 5). These results highlight the contribution of each component to the thermal behavior of the blends and demonstrate the effect of both composition and processing technology on the thermal properties of the materials.

### 3.1. Comparison of Technologies

A comparison between the two manufacturing technologies was carried out. While previous studies have already reported comparisons between FFF and FGF using pure polymers [6,7], the present analysis focused on the polymer blend and its influence on the manufacturing process. It is of interest to evaluate how the blend behaved during extrusion and deposition, as well as to identify any differences in the final properties of the printed specimens attributable to the printing technology used. To this end, the mean values obtained from the tests performed on the samples described in Section 2 were analyzed.

#### 3.1.1. Tensile Test of FFF and FGF Specimens

During the tensile test, no clear visual differences were observed in either the visual inspection or the dimensional comparison of rectangular specimens manufactured via FFF and FGF, as both printing technologies were able to accurately reproduce the rectangular specimen geometry. Then, specimens were subjected to tensile loading until failure, showing a similar mechanical behavior (Table 7). The PDOPCL blend exhibited a high degree of plasticity, with elongations higher than 500% until break, highlighting the ability to support large strains prior to failure in both cases. The elastic modulus (E_t_) of the FFF-printed specimens was 131 N, compared to 134 N for the FGF-printed specimens, indicating a similar performance of both printing technologies. During the test, all specimens exhibited a stress-induced whitening (Figure 6, right) as they were stretched, which has been previously reported in the literature for materials such as PCL [26] and is indicative of the microstructural damage and molecular rearrangements that occur under tensile loading, which is commonly associated with semicrystalline polymers [27].

#### 3.1.2. Radial Strength

Regarding the PDOPCL tubular specimens manufactured using the two printing technologies, visual differences were observed (Figure 7). Although the same printing temperature (150 °C) and nozzle diameter (0.8 mm) were used in both cases, and despite the difference in extrusion widths defined in the slicer software (1.0 mm for FFF and 0.96 mm for FGF), the FFF specimens exhibited a lower wall thickness (0.8 mm) in comparison to the FGF specimens (average thickness of 1.2 mm).

As spiral mode was used to print in both groups, this observation is likely attributable to the higher extrusion multiplier employed in the FGF printer (value of 5.0, see Table 6). This factor may also explain the differences in surface finishing, as the layers of the FFF specimens were visible, whereas the FGF specimens exhibited a more homogeneous appearance, with the material showing more melting during deposition, resulting in reduced visibility of individual layer lines.

Regarding the mechanical behavior, both groups exhibited localized white regions, as before. Although they were less visibly affected (polymers were not studied until break in this test), white regions confirm that clear internal stresses were generated during radial compression.

Concerning the maximum forces recorded, the FFF-printed specimens exhibited a higher maximum radial compression force (481.46 N) than the FGF-printed specimens (414.03 N). Some differences in dimensions have been identified, as reported in Table 8. Since slight differences in specimen dimensions were identified (Table 8), the results were normalized to enable comparison between both groups, as it has been identified that radial force is influenced by specimen diameter and length [28]. According to ASTM F3067, radial pressure is an area-normalized parameter that accounts for specimen diameter and length and can, therefore, be used to compare tubular structures of different dimensions.

The radial pressure (P) was calculated according to Equation (1), where $F_{R}$ is the radial force and *A* is the cylindrical area of the specimen:

(1) $$P=\frac{F_{R}}{A}.$$

Normalization of radial pressure was performed to compare specimens with different diameters and length. 

The area is described using Equation (2), where *D* is the external diameter and *L* is the length of the specimen:

(2) A=πDL.

Calculation of the area.

Using the dimensions reported in Table 8 and Equations (1) and (2), the mean radial pressure of the FFF specimens was 3.44 MPa, whereas the FGF specimens showed a mean radial pressure of 2.85 MPa, corresponding to a difference of approximately 21%.

However, it should be noted that radial pressure normalization according to ASTM F3067 only accounts for specimen diameter and length and does not account for wall thickness. Consequently, the observed differences cannot be attributed exclusively to the manufacturing technology, as wall thickness may also contribute to the measured response. The present results (Figure 8), therefore, demonstrate differences in the radial mechanical performance of the manufactured specimens rather than isolating the effect of the manufacturing process itself.

Notably, the higher radial pressure obtained for the FFF specimens was observed despite their lower wall thickness (0.8 mm versus 1.2 mm).

### 3.2. Comparison of Material for Airway Stent Application

On the other hand, the suitability of the blend investigated in this study for tubular airway-support applications manufactured by additive manufacturing techniques was evaluated in comparison to raw materials, studying the effect of the manufacturing technique in the final application and the possible application of the blend to tracheal stents. To ensure comparability of the materials regardless of the technology and, as raw materials were originally acquired as filaments, all specimens were manufactured using the FFF printer.

#### 3.2.1. Tensile Test of Different Materials

PDO exhibited the highest resistance to tensile loading, indicating the highest ultimate tensile strength (σ_M_) among the three materials studied. Moreover, PCL showed the highest ductility, reaching an elongation at break of over 1000%, which means it deforms approximately ten times its original length before failure. Regarding the PDO/PCL blend, an intermediate elastic modulus was observed (133 MPa), promoting a slightly higher mean elastic modulus with respect to pure PCL (121.77 MPa; Table 9, Figure 9). However, the blend exhibited a lower elongation at break that may be related to the limited miscibility between both polymers, as suggested in previous studies [29], leading to a poorest stress resistance, which may be caused because of tensions not being distributed homogeneously throughout the material.

#### 3.2.2. Radial Compression of Tubular Specimens and Stents

In the tubular group, PDO showed the highest radial strength (694.93 N), being the most suitable material for the application requiring radial support, whereas PDOPCL exhibited the poorest resistance to radial compression (447.75 N), with PCL showing an intermediate behavior (556.37 N; Figure 10).

As stents have a particular geometry due to the lattice structure of their walls, the same test was conducted fabricating AM stents, identifying the same tendency as before. The stent with the highest radial compression resistance was the PDO one (1099.87 N), followed by the PCL sample (764.01 N). Again, the stent of PDOPCL was the one showing the poorest resistance (517.56 N).

As the stents and the reference specimens had different diameters and lengths, radial force was normalized (Equations (1) and (2)) according to ASTM F3067. The resulting values were converted into radial pressure (P, in MPa) by dividing radial force (N) by the contact area (A), using Table 10 and Table 11. The comparison corroborated with the results of the previous tests, as similar trends were observed for all materials. In summary, PDO exhibited the highest resistance to radial compression, whereas the PDO/PCL blend showed the poorest mechanical performance. In general, the radial pressures obtained under radial compression for stents were lower than those measured for the tubular specimens. These results indicate that the mechanical response under radial compression is strongly dependent on the structural architecture. The lower strength observed for the X-designed stents may be associated with both the geometry itself and the layer-by-layer deposition characteristic of the FDM process. This information may be relevant for evaluating the influence of the manufacturing technology on complex geometries, since different results were obtained from the computational simulations [23]. This finding highlights the importance of considering process-dependent effects when predicting the mechanical behavior of additively manufactured structures.

## 4. Discussion

This study aimed to develop a novel material based on the blending of two polymers with desirable properties for tracheal stent applications, PDO and PCL. A key objective was to obtain material that can be directly processed by additive manufacturing without the use of additives, compatibilizers, or chemical modification steps. Furthermore, the study compared filament-fed and pellet-fed FDM technologies, providing insight into their respective capabilities for manufacturing polymer blends. In this way, the work is intended to create a relation between advanced multifunctional materials and their practical implementation in the healthcare industry, promoting more accessible and flexible manufacturing routes for personalized medical devices.

In this study, the comparison between FFF and FGF revealed that rectangular specimens exhibited similar mechanical behavior regardless of the manufacturing technology, whereas differences in radial mechanical performance of tubular specimens were observed between the FFF- and FGF-manufactured samples. However, these differences cannot be attributed exclusively to the manufacturing technology, as the specimen geometries were not identical. In the case of the tubular specimens, an initial assumption might be that the FGF-produced samples would exhibit higher mechanical resistance, as they showed a better surface finish, characterized by a larger deposited width and a reduced visibility of layer lines. However, the experimental results showed the opposite trend. Differences in the manufacturing processes and specimen geometry may have contributed to this behavior. A limitation of the present study is that, although the same geometry and slicing software were used for all specimens, differences in wall thickness were observed in the tubular specimens due to the inherent differences in material extrusion between the two manufacturing technologies, despite the independent optimization of printing speed, extrusion multiplier, and extrusion width for each system to achieve a similar flow rate. Consequently, different processing parameters were employed, which may have led to variations in the cooling time between successive polymer layers during printing. As both components of the blend are semicrystalline polymers and have been reported to exhibit limited miscibility [29], they generally exhibit slower cooling times than materials such as PLA [7,30,31] that have been extensively studied. When analyzing the printing processes, notable differences can be identified. In the FGF process, the pellets are heated at the entrance of the extrusion screw and remain at elevated temperature while being pushed along the screw until extrusion. Consequently, when the material is deposited through the nozzle, it has been exposed to the printing temperature (150 °C) for approximately 60 s. In contrast, during FFF printing, the filament is heated only as it passes through the hot end immediately before extrusion, remaining at elevated temperature for approximately 4 s, which is almost 1 min less than in the FGF process. Once the thermal exposure time of the material is known, it is possible to estimate the time required for the printer to deposit a new layer at the same location. This interval provides an approximation of the cooling time available to the polymer before the deposition of subsequent material. Since the same cooling fan configuration (100% fan speed) was used in both technologies, the cooling conditions can be considered comparable. For the rectangular specimens, the relatively large printing area resulted in a layer-to-layer interval of 29 s for FFF and 75 s for FGF, providing sufficient time for the deposited material to cool before the next layer was deposited. However, for the tubular specimens, spiral printing mode was employed, resulting in a continuous deposition process with only 15 s for FFF and 31 s for FGF between successive layers at the same location. Consequently, in the FGF process, the polymer is subjected to a longer heating time at elevated temperature and, in geometries where the cooling time between deposited layers is limited, this temperature may negatively affect the mechanical properties of the device.

When printers were compared, the ability of the geometry to undergo adequate cooling during fabrication had a greater impact on the final mechanical performance than whether the material was processed through filament-fed (FFF) or pellet-fed (FGF) extrusion, so when identical printing parameters were used, both technologies produced comparable results for larger samples. Consequently, the choice of technology can be made according to the specific situation. If filament material is already available, FFF technology requires very little calibration. Conversely, if the filament is not available, FGF technology can be an attractive alternative, as it avoids the time and manufacturing costs associated with filament production. Therefore, regarding the manufacturing technology, it can be concluded that the feeding method itself is not the primary factor influencing the mechanical properties of the printed components [7], showing a similar result, but it is the geometry of the factor that appears to have a more clear role, as it determines the cooling time available between successive layers [32].

On the other hand, regarding the materials, no universally accepted threshold values for radial force, radial stiffness, or radial stress have been defined in the literature for airway stents. However, the mechanical performance required for tracheal stent applications should be balanced to provide sufficient radial force to keep the airway open without causing tissue damage. This balance has been reported as a key requirement for suitable airway stent materials, including PCL and PDO [15,33]. At first glance, the PDOPCL blend may demonstrate an intermediate tensile response between PCL and PDO, with a slightly higher mean elastic modulus than pure PCL. In contrast, radial compression tests reveal that PDOPCL exhibited lower radial pressure than raw polymer. This finding suggests that, although blending PDO and PCL may be beneficial for tensile performance and applications such as sutures [34], the resulting blend does not provide the same advantages for applications where radial support is required [15,33], such as tracheal stents. It is worth mentioning that one limitation of this study is that the degradation of the polymers was not investigated [1,7]. As a result, the effect of the different printing parameters on the degradation behavior of the polymers remains unknown and should be addressed in future work.

Furthermore, this study highlights the importance of investigating the relationship between the manufacturing technology and the geometry of the device, as the geometry plays a critical role in key printing process parameters, such as polymer cooling time and interlayer adhesion [35], both of which directly influence the mechanical properties of the final component and, consequently, its in-service performance.

## 5. Conclusions

When assessing the suitability of a medical device, the entire workflow must be considered, including material selection, manufacturing, and in-service performance. Consequently, each application should be studied individually, as the optimal combination of each parameter may vary depending on the intended use of the device.

Regarding the material blend, the PDOPCL blend may be of interest for applications requiring improved elastic modulus in comparison to PCL. However, based on the radial compression results obtained using FFF-manufactured specimens, it may not be suitable for applications subjected to radial loading, such as stents, which were the primary target application considered in this study.

Moreover, the mechanical behavior of additively manufactured structures cannot be attributed solely to the material itself, but also to the interaction between the selected manufacturing process and the geometric characteristics of the printed part. Consequently, FFF and FGF provide similar results, and the technology can be selected based on practical requirements.

As a conclusion, the results of this study highlight that device performance is governed not only by the intrinsic properties of the materials but also by the interplay between manufacturing technology and geometry.

## Figures and Tables

**Figure 1:**
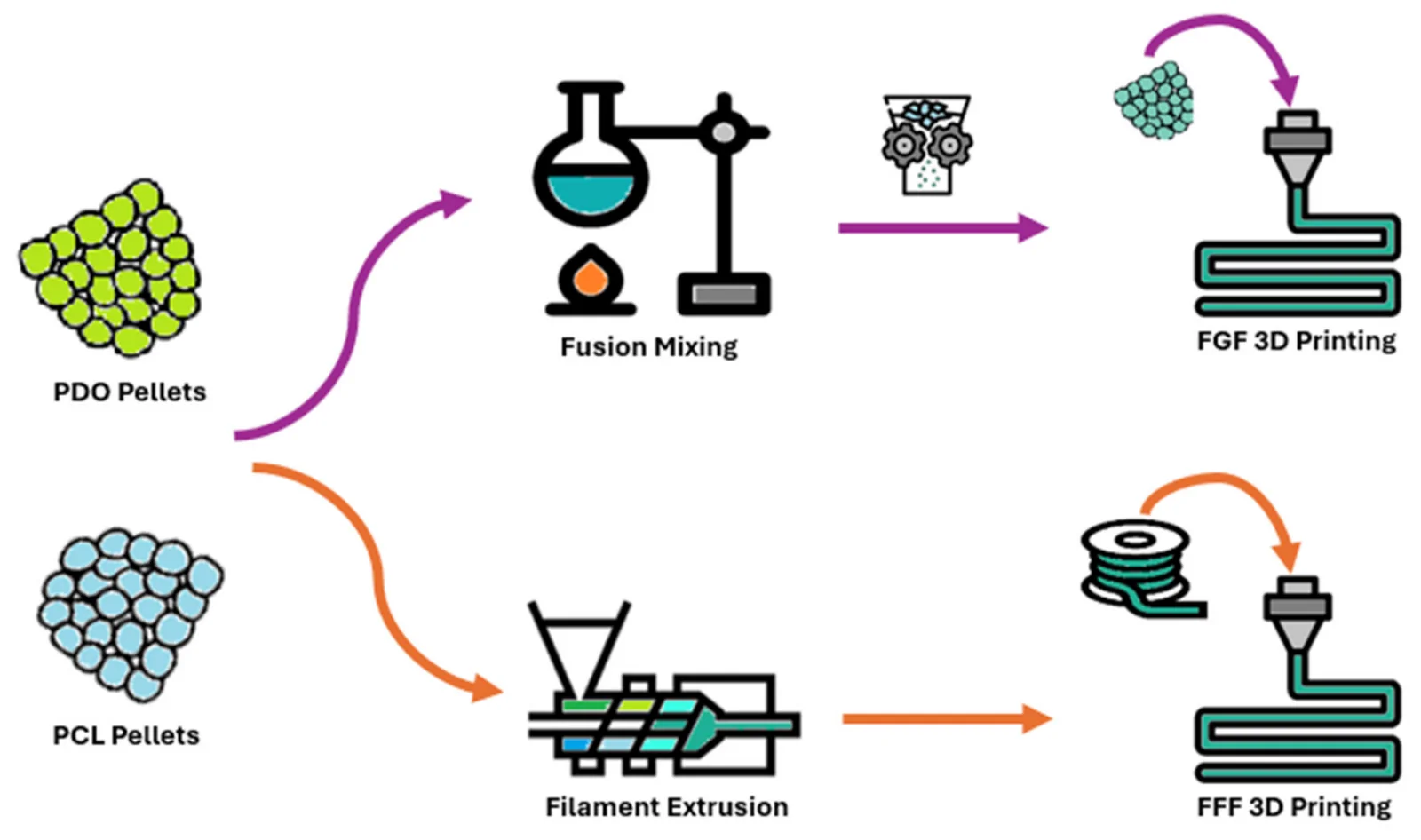
Processing workflow for PDO and PCL: FFF vs. FGF.

**Figure 2:**
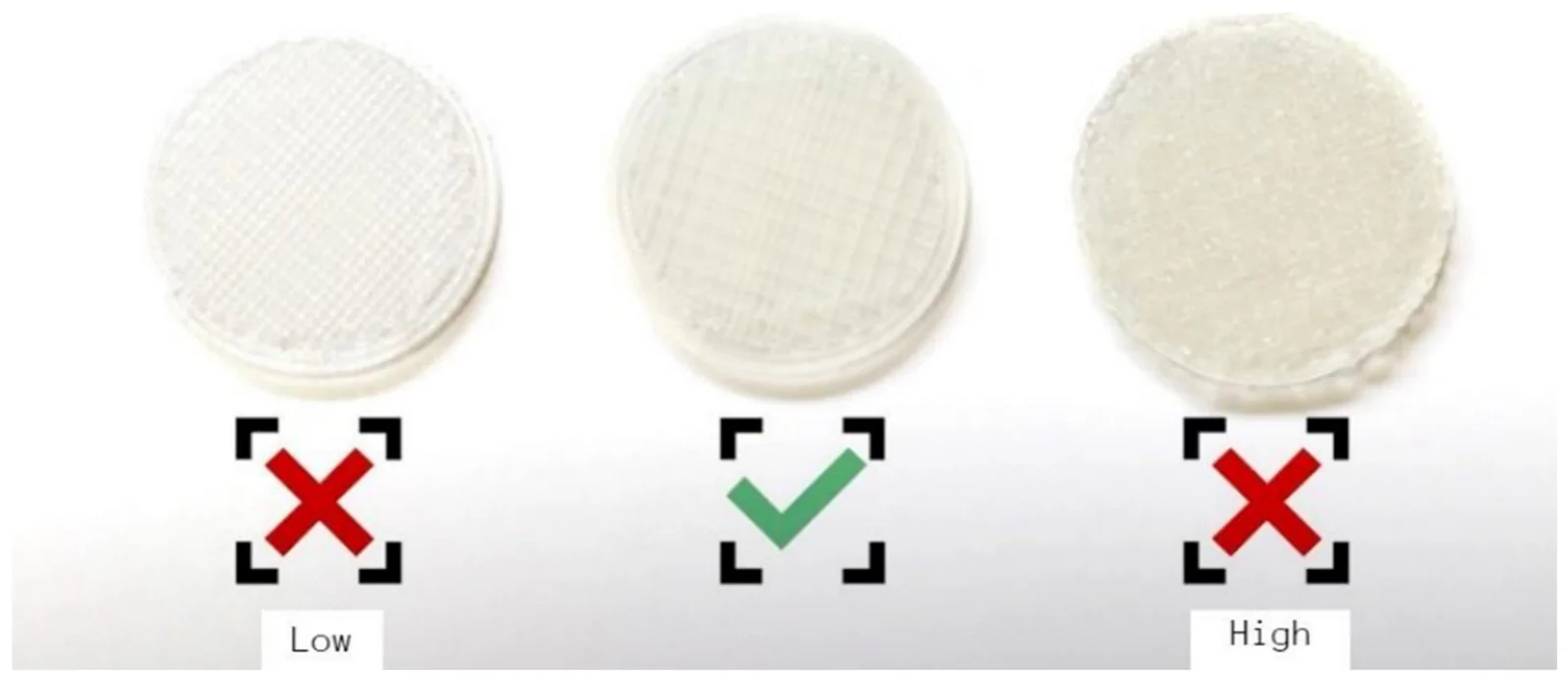
Calibration indications provided by Indart3D to adjust the flow rate of the FGF printer.

**Figure 3:**
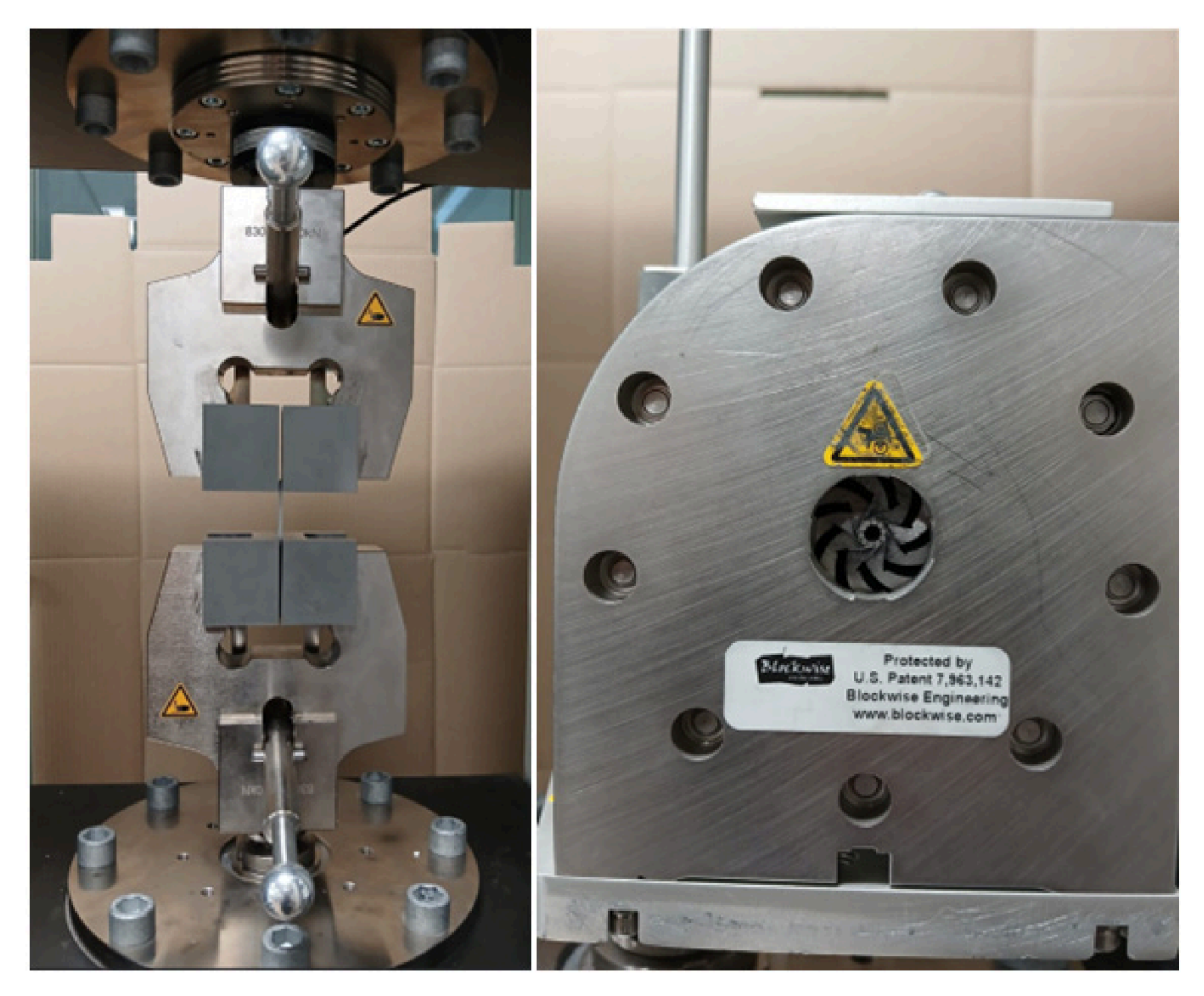
Test configuration for tensile (**left**) and radial (**right**) compression using the Zwick Roell universal testing machine.

**Figure 4:**
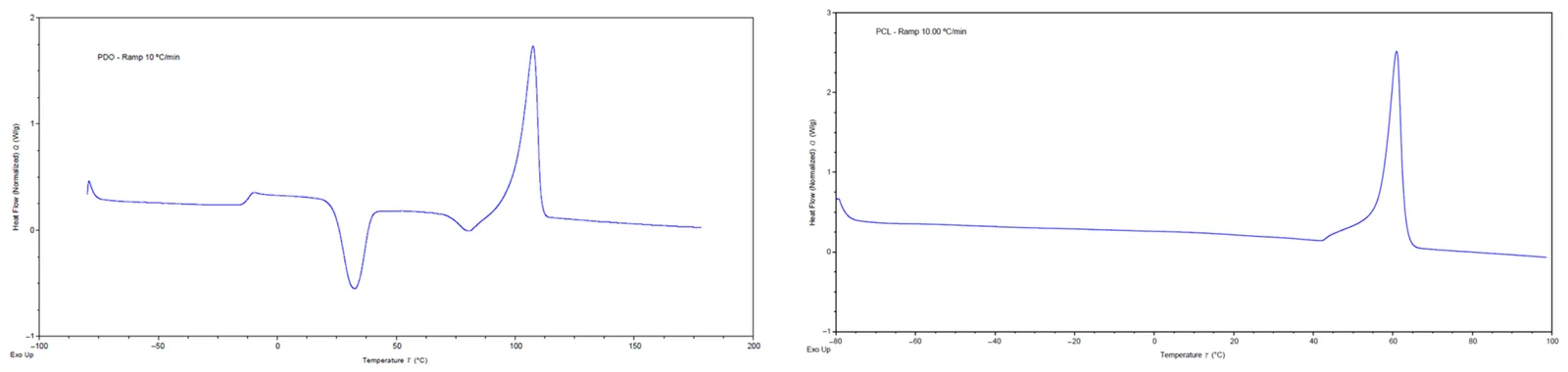
DSC for PDO (**left**) and PCL (**right**).

**Figure 5:**
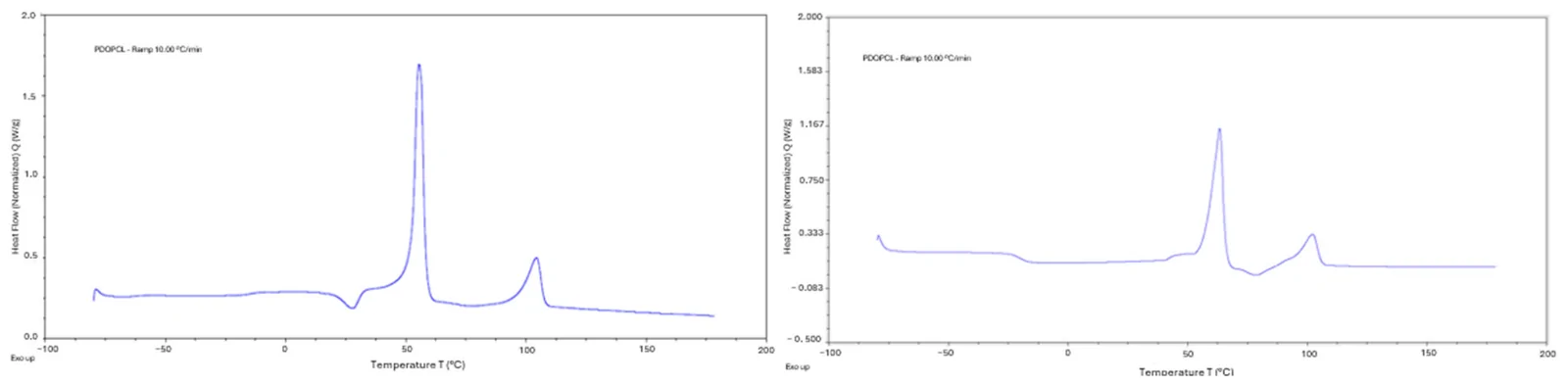
DSC of the PDOPCL blend in filament form (**left**) and pellet form (**right**).

**Figure 6:**
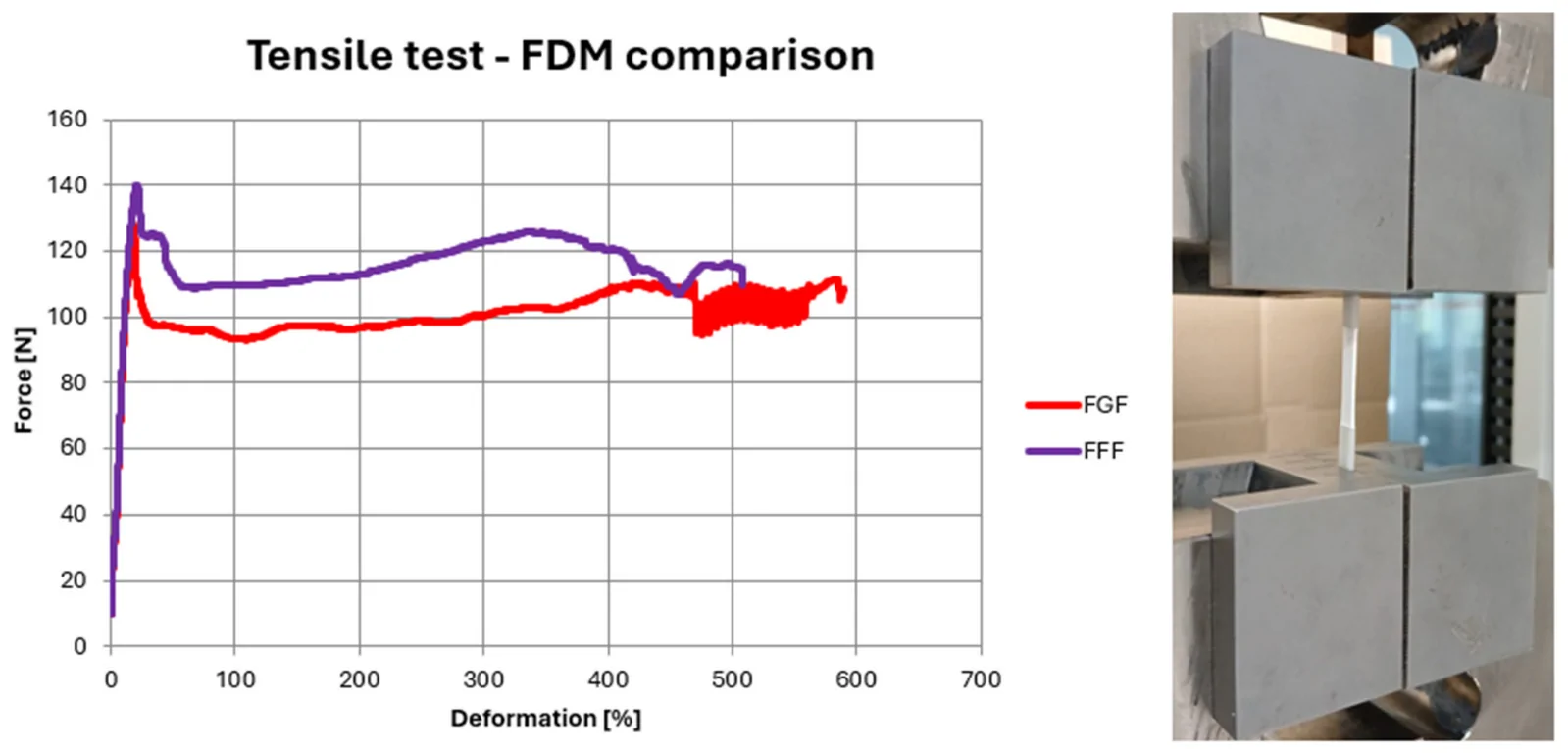
(**Left**) Tensile test results for FGF and FFF. (**Right**) White regions due to stress concentration. Results are reported as the means of 5 samples per group.

**Figure 7:**
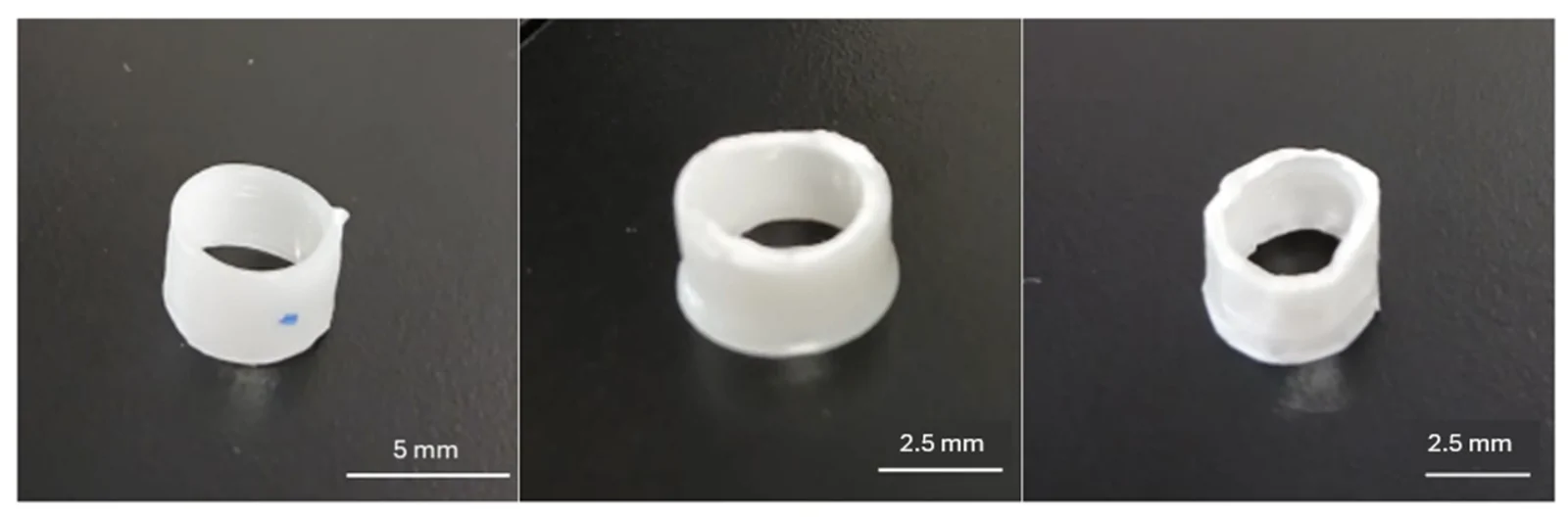
(**Left**) Identification of visible layer lines in the FFF specimen. (**Center**) FGF specimen before mechanical testing, with a better surface finish. (**Right**) The same FGF specimen but after testing, showing the appearance of stress-induced whitening and permanent deformation.

**Figure 8:**
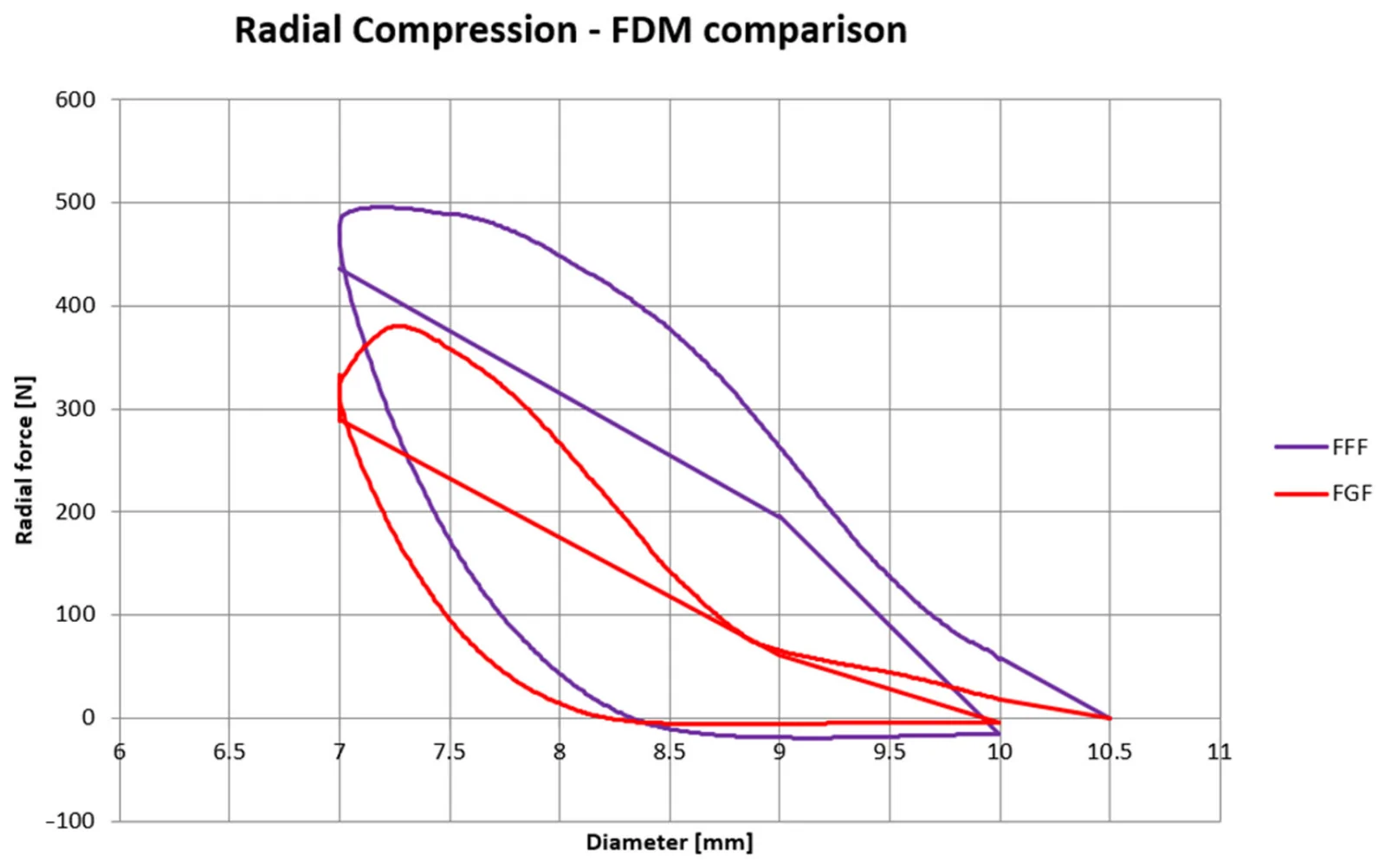
Radial compression test of FFF and FGF tubular specimens. Results are reported as the means of 5 samples per group.

**Figure 9:**
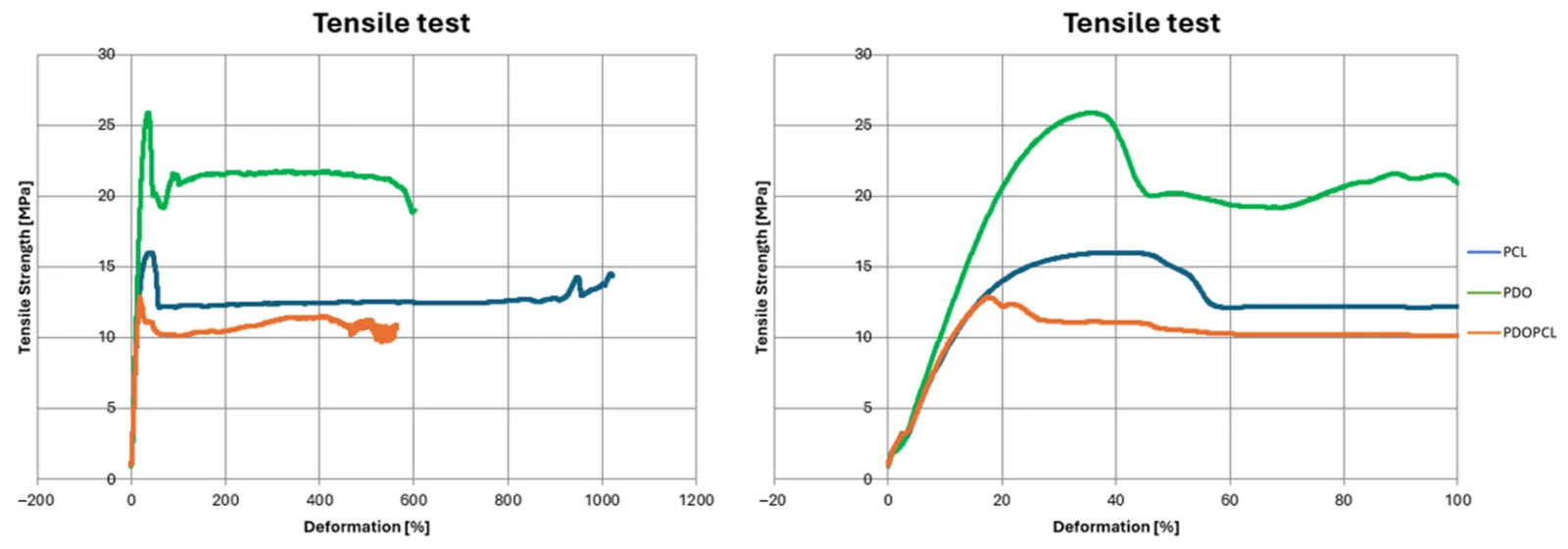
Tensile test for FFF specimens. (**Left**) Complete test representation. (**Right**) Detail of the elastic region of the figure on the left. Results are reported as the means of 5 samples per group.

**Figure 10:**
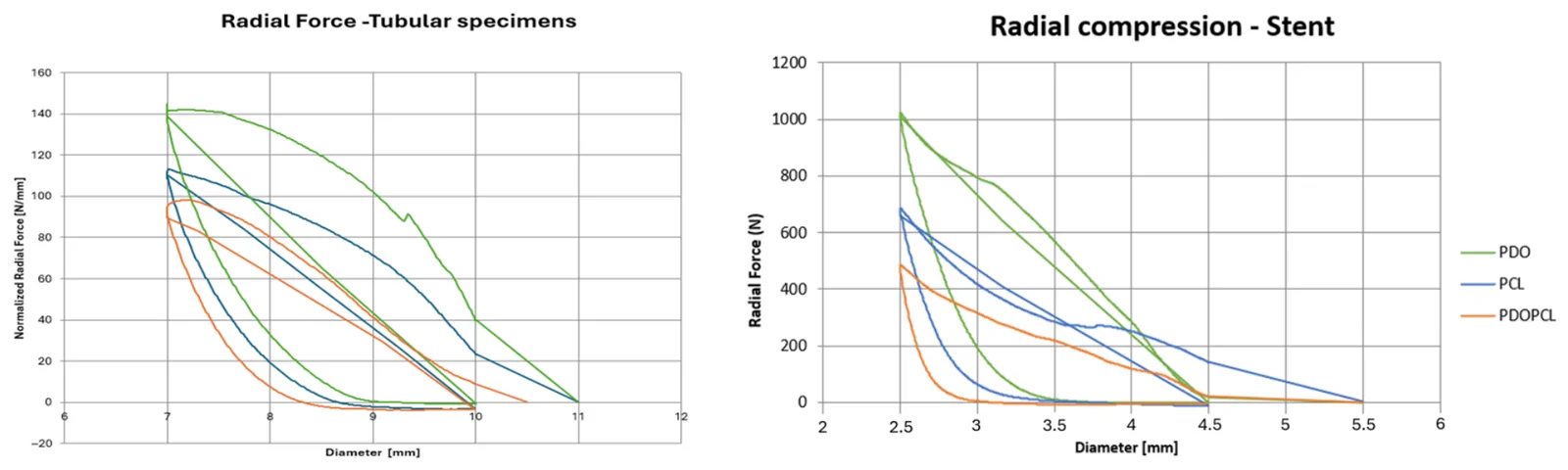
Radial compression test for the three materials under investigation. Results are reported as the means of 5 samples for tubular specimens and 3 samples of each material of stents.

**Table 1:** Key temperature parameters for PDO and PCL, provided by the manufacturers.

	PDO	PCL
Melting point	95–110 °C	58–60 °C
Decomposition temperature	250 °C	200 °C

**Table 2:** Drying condition for filaments.

Material	Temperature (°C)	Time (h)
PDO	45	24
PCL	35	24
PDOPCL	40	24

**Table 3:** Configuration of parameters of the filament production process using the Filament Maker Composer 450 extrusion system.

	H1 (°C)	H2 (°C)	H3(°C)	H4 (°C)	Fan (%)	RPM
PDOPCL	100	112	128	123	100	2

**Table 4:** Printing parameters of the PDOPCL blend using the FGF printer.

	Zona Input	Zona Output	Flow Rate
PDOPCL	100 °C	150 °C	250

**Table 5:** Comparison of printing parameters for both technologies using PDOPCL.

	FFF	FGF
Nozzle diameter (mm)	0.8	0.8
Layer height (mm)	0.5	0.5
Nozzle temperature (°C)	150	150
Bed temperature (°C)	45	45
Chamber temperature (°C)	25	25
Print speed (mm/s)	10	2
Extrusion width	1.0	0.96
Extrusion multiplier	1.0	5.0

**Table 6:** Printing parameters for the stents using the FFF printer.

	PCL	PDO	PDOPCL
Nozzle diameter (mm)	0.25	0.25	0.25
Nozzle temperature (°C)	130	200	150
Bed temperature (°C)	45	45	45
Chamber temperature (°C)	25	25	25
Print speed (mm/s)	20	30	20
Retraction speed (mm/s)	40	40	40
Retraction distance (mm)	0.4	0.4	0.4

**Table 7:** Comparison of mechanical properties obtained from tensile testing. The E_t_, the strain at module strength (ε_M_), and the strain at break (ε_B_) are represented. Results are reported as mean ± standard deviation, using 5 samples per group.

PDOPCL	E_t_	ε_M_	ε_B_
	MPa	%	%
FFF	131.11 ± 36.76	25.14 ± 1.14	540.07 ± 125.03
FGF	134.93 ± 28.46	25.12 ± 8.09	574.43 ± 190.21

**Table 8:** Average dimensions of tubular specimens. Results are reported as mean ± standard deviation, using 5 samples per group.

	Diameter	Height
	mm	mm
FGF	9.35 ± 0.81	4.94 ± 0.4
FFF	9.35 ± 0.15	4.77 ± 0.23

**Table 9:** Tensile test for FFF specimens. E_t_, σ_Y_ (yield stress), σ_M_, ε_M_, σ_B_ (breaking stress), and ε_B_ are represented. Results are reported as mean ± standard deviation, using 5 samples per group.

	E_t_	σ_Y_	σ_M_	ε_M_	σ_B_	ε_B_
	MPa	MPa	MPa	%	MPa	%
PCL	121.77 ± 35.82	3.26 ± 0.28	16.05 ± 0.43	442.56 ± 1.14	14.47 ± 0.67	1029.58 ± 19.56
PDO	151.90 ± 30.16	2.34 ± 0.73	28.48 ± 1.46	36.41 ± 0.93	22.01 ± 8.36	673.39 ± 29.41
PDOPCL	133.02 ± 25.31	3.13 ± 0.37	15.77 ± 3.19	25.13 ± 4.99	8.65 ± 3.19	557.25 ± 168.13

**Table 10:** Average dimensions of tubular specimens and stents fabricated by FFF. Results are reported as mean ± standard deviation. Five tubular specimens and three stents per material were analyzed.

	Specimens		Stent	
	Diameter	Length	Diameter	Length
	mm	mm	mm	mm
PCL	9.48 ± 0.34	4.88 ± 0.1	4.3 ± 0.06	39.87 ± 0.03
PDO	9.66 ± 0.06	4.92 ± 0.06	4.35 ± 0.08	39.97 ± 0.07
PDOPCL	9.35 ± 0.15	4.77 ± 0.22	4.36 ± 0.11	39.94 ± 0.08

**Table 11:** Comparison of normalized radial pressure between geometries and materials. Results are reported as mean ± standard deviation. Five tubular specimens and three stents of each material were analyzed.

Radial Pressure (P)	Specimens (MPa)	Stents (MPa)
PCL	3.83 ± 0.16	1.42 ± 0.02
PDO	4.65 ± 0.08	2.01 ± 0.04
PDOPCL	3.2 ± 0.19	0.95 ± 0.03

## Data Availability

The original contributions presented in this study are included in the article. Further inquiries can be directed to the corresponding author.

## Abbreviations

The following abbreviations are used in this manuscript:

FDM	Fused deposition modeling
AM	Additive manufacturing
PCL	Polycaprolactone
PDO	Polydioxanone
FFF	Fused filament fabrication
FGF	Fused granular fabrication
DSC	Differential scanning calorimetry
Tm	Melting temperature
E_t_	Elastic modulus
ε_M_	Strain at elastic modulus
ε_B_	Strain at break
σ_M_	Ultimate tensile strength
σ_Y_	Yield stress
σ_B_	Breaking stress
